# Four weeks of high‐intensity training in moderate, but not mild hypoxia improves performance and running economy more than normoxic training in horses

**DOI:** 10.14814/phy2.14760

**Published:** 2021-02-21

**Authors:** Kazutaka Mukai, Hajime Ohmura, Yuji Takahashi, Yu Kitaoka, Toshiyuki Takahashi

**Affiliations:** ^1^ Equine Research Institute Japan Racing Association Shimotsuke Japan; ^2^ Kanagawa University Yokohama Kanagawa Japan

**Keywords:** horse, hypoxic training, performance, running economy

## Abstract

We investigated whether horses trained in moderate and mild hypoxia demonstrate greater improvement in performance and aerobic capacity compared to horses trained in normoxia and whether the acquired training effects are maintained after 2 weeks of post‐hypoxic training in normoxia. Seven untrained Thoroughbred horses completed 4 weeks (3 sessions/week) of three training protocols, consisting of 2‐min cantering at 95% maximal oxygen consumption $\left(\dot{V}O_{2\max}\right)$ under two hypoxic conditions (*H16*, *F*_I_O_2_ = 16%; *H18*, *F*_I_O_2_ = 18%) and in normoxia (*N21*, *F*_I_O_2_ = 21%), followed by 2 weeks of post‐hypoxic training in normoxia, using a randomized crossover study design with a 3‐month washout period. Incremental treadmill tests (IET) were conducted at week 0, 4, and 6. The effects of time and groups were analyzed using mixed models. Run time at IET increased in *H16* and *H18* compared to *N21*, while speed at $\dot{V}O_{2\max}$ was increased significantly only in *H16*. $\dot{V}O_{2\max}$ in all groups and cardiac output at exhaustion in *H16* and *H18* increased after 4 weeks of training, but were not significantly different between the three groups. In all groups, run time, $\dot{V}O_{2\max}$, $V_{\dot{V}O2\max}$, ${\dot{Q}}_{\max}$, and lactate threshold did not decrease after 2 weeks of post‐hypoxic training in normoxia. These results suggest that 4 weeks of training in moderate (*H16*), but not mild (*H18*) hypoxia elicits greater improvements in performance and running economy than normoxic training and that these effects are maintained for 2 weeks of post‐hypoxic training in normoxia.

## Graphical abstract

The present study demonstrates that 4 weeks of training in moderate (*F*_I_O_2_ = 16%), but not mild hypoxia (*F*_I_O_2_ = 18%) was sufficient to elicit greater improvements in performance and running economy than normoxic training and that the effects of the hypoxic training were maintained over 2 weeks of post‐hypoxic training. Although trainers should monitor weight loss, our results can give a new insight into hypoxic training in horses and provide a new strategy for training programs in Thoroughbred racehorses.

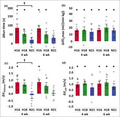

## 1 INTRODUCTION

Altitude/hypoxic training is popular in endurance athletes and has been used recently in middle‐distance runners, swimmers, and speed skaters. Although the efficacy of altitude/hypoxic training for sea‐level exercise performance remains controversial from a research perspective, athletes continue to use it to train for competitions. Most commonly, athletes both live and train at moderate to high altitude (live high‐train high, LHTH) or live at altitude and train at sea level (live high‐train low, LHTL). Previous reports and reviews have shown increases in exercise performance, maximal oxygen consumption $\left(\dot{V}O_{2\max}\right)$, and hemoglobin mass after several weeks of LHTH and/or LHTL training (Bonetti & Hopkins, 2009; Millet et al., 2010; Robertson et al., 2010).

Another hypoxic training program gaining popularity is the live low‐train high (LLTH) model. This model involves athletes living in normoxia and performing some training sessions in hypoxia. While several LLTH studies failed to demonstrate benefits in LLTH compared with equivalent normoxic training (McLean et al., 2014), some studies demonstrated that LLTH training can enhance exercise performance, maximal workload, and $\left(\dot{V}O_{2\max}\right)$ (Czuba et al., 2011), and can augment skeletal muscle mitochondrial density, capillary‐to‐fiber ratio, and fiber cross‐sectional area (Desplanches et al., 1993; Vogt et al., 2001), likely via up‐regulation of hypoxia‐inducible factor 1α (HIF‐1α)(Vogt et al., 2001). Some authors also suggest that LLTH may improve anaerobic exercise performance (Hamlin et al., 2010; Hendriksen & Meeuwsen, 2003), possibly via increases in muscle buffering capacity (Gore et al., 2001) and increased glycolytic enzyme activity (Puype et al., 2013).

When athletes and coaches use hypoxic training in practical situations, a key question is when is the best timing to return to sea level before a race to optimize performance. The general consensus among top coaches suggests that endurance performance is optimized after 14 days at sea level after altitude/hypoxic training (Dick, 1992), but there is limited scientific evidence to support this opinion. While some researchers suggest that repeated sprint ability and hemoglobin mass are higher at 3 weeks after hypoxic training compared with pre‐hypoxic training levels (Brocherie et al., 2015), another group reported that most hematological adaptations after altitude training are lost in 9 days (Pottgiesser et al., 2012). In addition, previous studies on the maintenance of post‐hypoxic training use mostly LHTL training and not LLTH.

Thoroughbred horses have high $\dot{V}O_{2\max}$, exceeding 180 mL/kg/min in trained individuals, and the aerobic contribution to total energy expenditure for a 120‐s sprint is estimated to reach >70% (Eaton et al., 1995; Ohmura et al., 2010). Furthermore, Thoroughbred horses have large amounts of glycogen (>600 mmol/kg dwt) in their muscle (Davie et al., 1999) and the lactate concentration in plasma and skeletal muscle during maximal exercise increases to more than 20 mmol/L and 20 mmol/kg, respectively (Kitaoka et al., 2014), which suggests that Thoroughbred horses also utilize the glycolytic pathway maximally for energy resources during high‐intensity exercise. Therefore, improvements in both aerobic and anaerobic capacity are needed to enhance equine racing performance. Previously, we reported that high‐intensity training (100% $\dot{V}O_{2\max}$ for 3 min, 3 sessions/week for 4 weeks) in moderate hypoxia (15% O_2_) improves run time and $\dot{V}O_{2\max}$ at incremental exercise tests (IET) in normoxia to a greater extent than the same training in normoxia (Mukai et al., 2020), which indicates that hypoxic training may be a strong strategy for better exercise performance without increasing absolute training speed and/or distance. However, very few studies have examined hypoxic training in horses (Davie et al., 2017; Ohmura et al., 2017), and further studies are needed to determine the optimal severity of hypoxia and the intensity, duration, and volume of training in hypoxia.

The purpose of this study was to investigate the hypothesis that horses trained in moderate and mild hypoxia for 4 weeks experience greater improvements in performance and aerobic capacity compared with horses trained in normoxia. In addition, we examined whether acquired training effects are maintained after 2 weeks of post‐hypoxic training in normoxia.

## 2 MATERIALS AND METHODS

Protocols for the study were reviewed and approved by the Animal Welfare and Ethics Committee of the Japan Racing Association (JRA) Equine Research Institute (Permit number: 2017–1, 2018–1). All surgery was performed under sevoflurane anesthesia and all incisions for catheter placements were performed under local anesthesia using lidocaine. All efforts were made to minimize animal suffering.

### 2.1 Horses

Seven untrained Thoroughbreds (2 geldings and 5 females; mean ±SE age, 7.9 ± 0.7 years; body weight, 512 ± 11 kg at the onset of the study) were used in this study. Each horse had a carotid artery moved surgically from the carotid sheath to a subcutaneous location under sevoflurane anesthesia to facilitate arterial catheterization. After recovery from surgery, the horses were trained to run on a treadmill (Sato I, Sato AB, Uppsala, Sweden) while wearing an open‐flow mask (Pascoe et al., 1999). After surgery, each horse was kept in a 17 x 22 m yard for approximately 6 h/day every day for at least 4 months before treadmill experiments began. All horses received 1 kg of oats, 1 kg of pelleted feed, and 3 kg of timothy hay in the morning, and 1 kg of oats, 2 kg of pelleted feed, and 3 kg of timothy hay in the afternoon. Water was available *ad libitum* during the study.

### 2.2 Experimental design

In a randomized crossover design, horses were trained in moderate hypoxia (*H16*; 16% inspired O_2_), mild hypoxia (*H18*; 18% inspired O_2_), or normoxia (*N21*; 21% inspired O_2_) for 3 days/week on a treadmill at a 6% incline. The horses were pastured in 17 × 22 m yards for approximately 6 h/day and walked for 1 h/day in a walker on the other 4 days during the training period. The training session consisted of a warm‐up (walking at 1.7 m/s for 30 min and trotting at 4 m/s for 2 min), cantering at 7 m/s for 1 min and for 2 min at the speed previously determined to elicit 95% $\dot{V}O_{2\max}$ measured in normoxia, followed by a cool‐down (1.7 m/s for 30 min) in all groups. In hypoxic groups, horses wore an open‐flow mask after walking for 30 min and were exposed to hypoxia during trotting for 2 min and cantering at 7 m/s for 1 min and at 95% $\dot{V}O_{2\max}$ for 2 min. After 4 weeks of hypoxic/normoxic training, all groups continued the same training protocols in normoxia for 2 weeks. Each training period was separated by 3 months to ensure a sufficient detraining interval.

### 2.3 Incremental exercise tests (IET) in normoxia

Incremental exercise tests in normoxia were conducted at weeks 0, 4, and 6. The procedure for the incremental exercise test, including oxygen consumption measurements and blood sampling, has been described previously (Mukai et al., 2017). Briefly, after catheters and transducers were connected and tested, the horse began its exercise. The horse warmed up by trotting at 4 m/s for 3 min, then cantering up a 6% incline for 2 min each at 1.7, 4, 6, 8, 10, 12, 13, and 14 m/s until the horse could not maintain its position at the front of the treadmill with humane encouragement. This condition was defined as exhaustion. Run time to exhaustion was measured with a stopwatch. For each speed, the horse ran on the treadmill for 90 s to allow the O_2_ transport system to come to steady‐state (equine $\dot{V}O_{2}$ comes to steady‐state faster than human $\dot{V}O_{2}$ does), then $\dot{V}O_{2}$ was calculated for the final 30 s of each step. Heart rate was recorded using a commercial heart rate monitor (S810, Polar, Kempele, Finland) and mean heart rate was calculated for the final 30 s of each step.

### 2.4 Oxygen consumption

Horses wore an open‐flow mask on the treadmill through which a rheostat‐controlled blower drew air. Air flowed through 25‐cm diameter tubing and across a pneumotachograph (LF‐150B, Vise Medical, Chiba, Japan) connected to a differential pressure transducer (TF‐5, Vise Medical) to ensure that bias flows during measurements were identical to those used during calibrations. Bias flow was set to keep changes in O_2_ concentration and CO_2_ concentrations at <1.5% to avoid having the horses rebreathe CO_2_. Oxygen and CO_2_ concentrations were measured with an O_2_ and CO_2_ analyzer (MG‐360, Vise Medical), and calibrations were used to calculate rates of O_2_ consumption and CO_2_ production with mass flowmeters (CR‐300, Kofloc, Kyoto, Japan) using the N_2_‐dilution/CO_2_‐addition mass‐balance technique (Fedak et al., 1981). Gas analyzer and mass flowmeter outputs were also recorded on personal computers using commercial hardware and software (DI‐720 and Windaq Pro+, DATAQ, Akron, OH) sampling at 200 Hz.

### 2.5 Blood sampling

Before leading a horse onto the treadmill, an 18‐gauge catheter (Surflow, Terumo, Tokyo, Japan) was placed in the horse's left carotid artery, and an 8‐F introducer (MO95H‐8, Baxter International, Deerfield, IL) was placed in the right jugular vein. A Swan‐Ganz catheter (SP5107 U, Becton, Dickinson and Company, Franklin Lakes, NJ) was passed via the jugular vein so that its tip was positioned in the pulmonary artery, confirmed by measuring pressure at its tip with a pressure transducer (P23XL, Becton, Dickinson and Company, Franklin Lakes, NJ). Mixed‐venous blood samples were drawn from the tip of the Swan‐Ganz catheter and arterial samples from the 18‐gauge carotid catheter into heparinized syringes at timed intervals for the final 30 s of each step and at 1, 3, and 5 min after exhaustion. Samples were stored on ice until measured immediately following the experiment. Blood samples were analyzed with a blood gas analyzer (ABL800 FLEX, Radiometer, Copenhagen, Denmark) and O_2_ saturation (*S*O_2_) and concentration (*C*O_2_) were determined using a hemoximeter (ABL80 FLEX‐CO‐OX, Radiometer, Copenhagen, Denmark). Following measurement of blood gases and oximetry, the blood was sampled for plasma lactate concentration using a lactate analyzer (Biosen S‐Line, EKF‐diagnostic GmbH, Barleben, Germany) after being centrifuged at 1870 × g for 10 min. The Swan–Ganz catheter in the pulmonary artery was connected to a cardiac output computer (COM‐2, Baxter International, Deerfield, IL) so that its thermistor registered pulmonary arterial temperature, could be recorded at each blood sampling and used to correct the blood gas measurements.

### 2.6 Hypoxic training protocol and measurements during exercise in the first week of each training period

The procedure for producing the hypoxic condition was slightly modified from the method previously described (Ohmura et al., 2010). Briefly, a mixing chamber was connected to the upstream flexible tube on a 25‐cm diameter open‐flow mask through which a flow of N_2_ was blown into the upstream end of the flow system and mixed with a bias‐flow of air of 80–120 L/s to create the desired inspired O_2_ concentration. Nitrogen gas flow was controlled with a mass flow meter (Model DPM3, Kofloc, Kyoto, Japan) connected to compressed gas cylinders through a gas manifold. Nitrogen gas flow was adjusted to maintain 16% or 18% O_2_ by monitoring the O_2_ concentration in the downstream arm of the mass flowmeter with an O_2_ analyzer (LC‐240UW, Vise Medical, Chiba, Japan) when horses ran in hypoxia.

In the first week of training for all groups, we collected arterial blood samples in the final 15 s of cantering at 95% $\dot{V}O_{2\max}$ during the exercise session to measure arterial blood gas variables (ABL800 FLEX and ABL80 FLEX‐CO‐OX, Radiometer, Copenhagen, Denmark) and plasma lactate concentration (Biosen S‐Line, EKF‐diagnostic GmbH, Barleben, Germany). We also recorded heart rate (S810, Polar, Kempele, Finland) during cantering.

### 2.7 Statistical analysis

Data are presented as mean ± standard error (SE). Differences in the variables between *H16*, *H18*, and *N21* during training sessions in the first week were analyzed using mixed models with a group as a fixed effect and horse as a random effect. Post hoc testing was performed by Tukey's test. After training, the with‐in subject changes were analyzed using mixed models for differences between groups with a group as a fixed effect and horse as a random effect. Tukey's tests were used as post hoc tests.

Pearson correlation was used to determine the relationship between the changes in the run time and body weight at IET after training and arterial O_2_ saturation (*S*_a_O_2_), peak plasma lactate concentration, and heart rate during exercise sessions in the first week of training periods. Statistical analyses were performed with commercial software (JMP 13.1.0, SAS Institute Inc, Cary, NC) with significance defined as *p* < 0.05.

## 3 RESULTS

### 3.1 Blood gas variables, heart rate, and plasma lactate concentration during exercise sessions in the first week of training

*S*_a_O_2_ was lowest at the last 15 s of a 2‐min run at 95% $\dot{V}O_{2\max}$ in *H16* and highest in *N21* (*p* < 0.0001, Table 1). Arterial O_2_ partial pressure (*P*_a_O_2_) in *H16* and *H18* was lower than that in *N21* (*p* < 0.0001, Table 1), and arterial carbon dioxide partial pressure (*P*_a_CO_2_) in *H16* and *H18* was higher than that in *N21* during exercise (*p* = 0.0013, Table 1). There were no differences in heart rate at the last 15 s of a 2‐min run at 95% $\dot{V}O_{2\max}$ between all groups (*p* = 0.96, Table 1). Arterial pH of *H16* was lower than that of *N21* (*p* = 0.0038, Table 1), and peak plasma lactate concentration of *H16* was higher than that of *H18* (*p* = 0.032, Table 1).

**TABLE 1:** Parameters on aerobic capacity and blood gas analysis during the exercise session at the 1^st^ week of training

	*H16*	*H18*	*N21*
*S*_a_O_2_ (%)	66.5 ± 1.7 ^a^	74.1 ± 1.7 ^b^	90.9 ± 1.3 ^c^
*P*_a_O_2_ (Torr)	38.8 ± 0.6 ^a^	44.8 ± 2.4 ^a^	68.8 ± 3.3 ^b^
*P*_a_CO_2_ (Torr)	59.1 ± 1.5 ^a^	55.3 ± 3.5 ^a^	42.3 ± 1.3 ^b^
Heart rate (bpm)	203 ± 4 ^a^	202 ± 3 ^a^	202 ± 4 ^a^
Arterial pH	7.210 ± 0.015 ^a^	7.247 ± 0.017 ^ab^	7.281 ± 0.011 ^b^
Peak lactate (mmol/L)	22.3 ± 2.7 ^a^	17.7 ± 1.4 ^b^	18.5 ± 1.0 ^ab^

Arterial O_2_ saturation (*S*_a_O_2_), arterial O_2_ partial pressure (*P*_a_O_2_), arterial carbon dioxide partial pressure (*P*_a_CO_2_), heart rate, arterial pH, and peak plasma lactate concentration. Values are means ± SE for 7 horses. Different letters indicate significant differences between groups (*p* < 0.05).

### 3.2 Effects of normoxic and hypoxic training on exercise performance and aerobic capacity at IET

**FIGURE 1:**
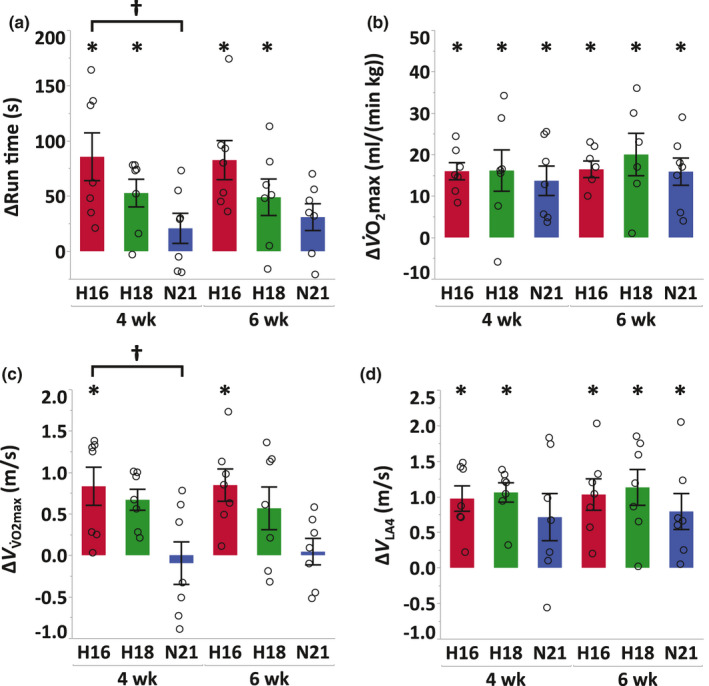
Changes in run time (a), $\dot{V}O_{2\max}$ (b), speed eliciting $\dot{V}O_{2\max}$ ($V_{\dot{V}O2\max}$; c) and speed at which plasma lactate concentration reached 4 mmol/L (*V*_LA4_; d) in IET from pre‐training to immediate post‐training (4 weeks) and 2 weeks of post‐hypoxic training in normoxia (6 weeks) either after moderate hypoxia (*H16*, red), mild hypoxia (*H18*, green), or normoxia (*N21*, blue). Values are mean ±SE. * Significant changes from pre‐training (*p* < 0.05). † Significant differences between groups (*p* < 0.05)

**FIGURE 2:**
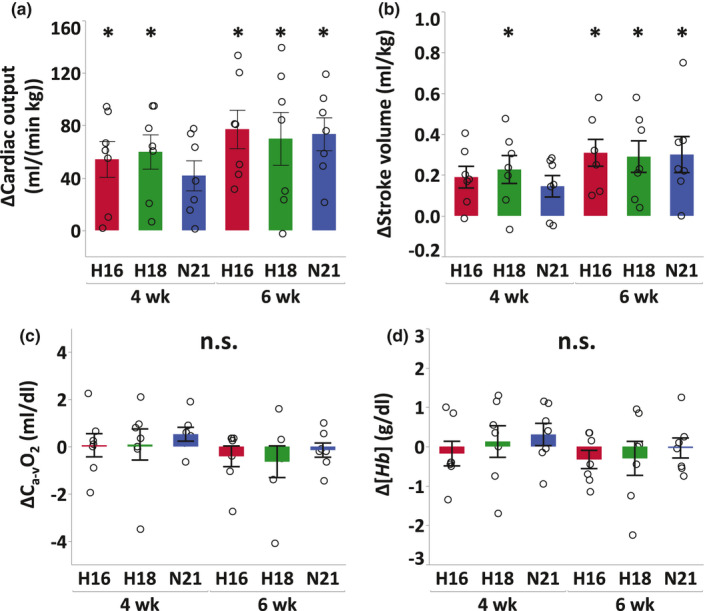
Changes in cardiac output (a), stroke volume (b), arterial‐mixed venous O_2_ difference (c), and hemoglobin concentration (d) at exhaustion in IET from pre‐training to immediate post‐training (4 weeks) and 2 weeks of post‐hypoxic training in normoxia (6 weeks) either after moderate (*H16*, red), mild hypoxia (*H18*, green), or normoxia (*N21*, blue). Values are mean ±SE. * Significant changes from pre‐training (*p* < 0.05). † Significant differences between groups (*p* < 0.05)

After 4 weeks of training, run time (*H16*, +20.6%, *p* < 0.0001; *H18*, +11.7%, *p* = 0.017) and maximal cardiac output (${\dot{Q}}_{\max}$: *H16*, +8.1%, *p* = 0.024; *H18*, +9.5%, *p* = 0.012) at IET increased in *H16* and *H18*, $\dot{V}O_{2\max}$ increased in all groups (*H16*, +9.8%, *p* = 0.0039; *H18*, +10.5%, *p* = 0.0025; *N21*, +8.8%, *p* = 0.025), and speed at $\dot{V}O_{2\max}$ $\left(V_{\dot{V}O2\max}\right)$ increased only in *H16* (+7.7%, *p* = 0.010)(Figure 1, Figure 2 and Table 2). Blood gas variables including hemoglobin concentration, O_2_ and CO_2_ partial pressures, and arterial‐mixed venous O_2_ concentration did not change in all groups during the training period (Figure 2 and Table 2). Changes in run time and $V_{\dot{V}O2\max}$ after 4 weeks of training were different between *H16* and *N21* (run time, *p* = 0.040; $V_{\dot{V}O2\max}$, *p* = 0.014), while the changes in $\dot{V}O_{2\max}$, ${\dot{Q}}_{\max}$, *SV*_max_, and blood gas variables were not different between the groups ($\dot{V}O_{2\max}$, *p* = 0.87; ${\dot{Q}}_{\max}$, *p* = 0.74; *SV*_max_, *p* = 0.99) (Figure 1 and Figure 2). Run time, $\dot{V}O_{2\max}$, $V_{\dot{V}O2\max}$, ${\dot{Q}}_{\max}$, *SV*_max_, and lactate threshold did not change after 2 weeks of post‐hypoxic training in normoxia in all groups compared with those at week 4 (Figure 1 and Table 2). Body weight decreased after 4 weeks of training in all groups (*H16*, −4.7%, *p* < 0.0001; *H18*, −4.1%, *p* < 0.0001; *N21*, −2.4%, *p* = 0.0003) and there was a significantly greater weight loss in *H16* compared to *N21* (*p* = 0.021), but not between *H18* and *N21*. These reductions in body weight lasted for 2 weeks after a switch to normoxic training (Table 2).

**TABLE 2:** Parameters on exercise performance, aerobic capacity, and blood gas analysis in normoxic incremental exercise tests at week 0, 4, and 6

	*H16*			*H18*			*N21*		
	0 week	4 weeks	6 weeks	0 week	4 weeks	6 weeks	0 week	4 weeks	6 weeks
Run time (s)	416 ± 26	501 ± 36 *	498 ± 36 *	438 ± 25	490 ± 34 *	487 ± 34*	433 ± 18	454 ± 25	464 ± 23
$\dot{V}O_{2\max}$ (mL/(min kg))	164 ± 3	180 ± 3 *	180 ± 4 *	161 ± 4	177 ± 3 *	175 ± 6 *	161 ± 4	175 ± 3 *	177 ± 3 *
Body weight (kg)	517 ± 11	493 ± 9 *	494 ± 10 *	518 ± 9	497 ± 11 *	498 ± 12 *	514 ± 10	502 ± 9 *	501 ± 8 *
$V_{\dot{V}O2\max}$ (m/s)	11.1 ± 0.4	12.0 ± 0.4 *	12.0 ± 0.4 *	11.5 ± 0.4	12.1 ± 0.4	12.0 ± 0.5	11.7 ± 0.2	11.6 ± 0.3	11.7 ± 0.3
${\dot{Q}}_{\max}$ (mL/(min kg))	666 ± 10	720 ± 19 *	743 ± 19 *	654 ± 16	713 ± 8 *	724 ± 15 *	662 ± 20	703 ± 11	735 ± 16 *
*SV*_max_ (mL/kg)	3.20 ± 0.08	3.39 ± 0.12	3.51 ± 0.13 *	3.13 ± 0.09	3.35 ± 0.07 *	3.42 ± 0.10 *	3.18 ± 0.13	3.32 ± 0.10	3.48 ± 0.14 *
*HR*_max_ (bpm)	209 ± 5	215 ± 5	213 ± 3	209 ± 4	213 ± 3	212 ± 4	209 ± 5	213 ± 4	214 ± 4
*V*_HRmax_ (m/s)	10.2 ± 0.4	10.9 ± 0.5	11.0 ± 0.4	10.4 ± 0.5	11.0 ± 0.4	11.1 ± 0.4	10.5 ± 0.3	10.6 ± 0.4	10.7 ± 0.4
*C*_a‐v_O_2_ (mL/dL)	24.6 ± 0.2	24.6 ± 0.2	24.2 ± 0.2	24.8 ± 0.2	24.8 ± 0.2	24.1 ± 0.2	24.3 ± 0.1	24.8 ± 0.2	24.1 ± 0.2
[*Hb*] (g/dL)	23.9 ± 0.5	23.7 ± 0.6	23.6 ± 0.6	24.0 ± 0.4	24.1 ± 0.5	23.7 ± 0.5	23.5 ± 0.5	23.8 ± 0.4	23.5 ± 0.6
*P*_a_O_2_ (Torr)	78.8 ± 0.7	79.0 ± 0.5	80.4 ± 1.0	83.0 ± 1.0	81.0 ± 1.0	79.2 ± 1.2	84.0 ± 0.9	79.8 ± 1.0	79.2 ± 0.8
*P*_v_O_2_ (Torr)	21.2 ± 0.1	19.7 ± 0.3	20.6 ± 0.3	22.3 ± 0.2	20.8 ± 0.2	21.0 ± 0.3	22.4 ± 0.2	19.9 ± 0.2	20.4 ± 0.3
*P*_a_CO_2_ (Torr)	52.9 ± 0.5	55.8 ± 0.6	55.0 ± 0.8	53.1 ± 0.6	56.7 ± 0.6	56.6 ± 0.6	52.9 ± 0.4	54.4 ± 0.6	54.7 ± 0.5
*P*_v_CO_2_ (Torr)	112.2 ± 1.6	126.7 ± 2.9	122.2 ± 2.2	110.9 ± 1.7	121.3 ± 2.2	120.0 ± 2.2	114.2 ± 1.7	119.5 ± 2.6	122.1 ± 2.2
*S*_a_O_2_ (%)	87.7 ± 0.4	86.5 ± 0.4	86.5 ± 0.5	88.9 ± 0.4	86.8 ± 0.5	85.9 ± 0.5	89.5 ± 0.4	87.5 ± 0.5	86.7 ± 0.4
*S*_v_O_2_ (%)	13.5 ± 0.3	11.7 ± 0.6	11.7 ± 0.4	14.9 ± 0.4	12.7 ± 0.5	12.5 ± 0.6	14.7 ± 0.5	12.5 ± 0.5	12.0 ± 0.5
pH_a_	7.207 ± 0.005	7.200 ± 0.009	7.203 ± 0.008	7.235 ± 0.006	7.218 ± 0.009	7.213 ± 0.010	7.227 ± 0.007	7.215 ± 0.010	7.220 ± 0.009
pH_v_	7.084 ± 0.006	7.070 ± 0.011	7.073 ± 0.008	7.101 ± 0.007	7.094 ± 0.010	7.073 ± 0.009	7.097 ± 0.011	7.085 ± 0.011	7.087 ± 0.009

Run time, maximal oxygen consumption ($\dot{V}O_{2\max}$), body weight, speed eliciting $\dot{V}O_{2\max}$ ($V_{\dot{V}O2\max}$), cardiac output (${\dot{Q}}_{\max}$), cardiac stroke volume (*SV*_max_), maximal heart rate (*HR*_max_), speed eliciting HR_max_ (*V*_HRmax_), arterial and mixed‐venous O_2_ difference (*C*_a‐v_O_2_), arterial and mixed‐venous O_2_ partial pressure (*P*_a_O_2_, *P*_v_O_2_), arterial and mixed‐venous carbon dioxide partial pressure (*P*_a_CO_2_, *P*_v_CO_2_), hemoglobin concentration ([*Hb*]), arterial and mixed venous O_2_ saturation (*S*_a_O_2_, *S*_v_O_2_), and arterial and mixed venous pH (pH_a_, pH_v_) at exhaustion during normoxic incremental exercise tests. Values are means ±SE for seven horses. *Significant changes from 0 week (*p* < 0.05).

### 3.3 Correlations between the variables during the exercise session and the changes of variables at IET after 4 weeks of training

**FIGURE 3:**
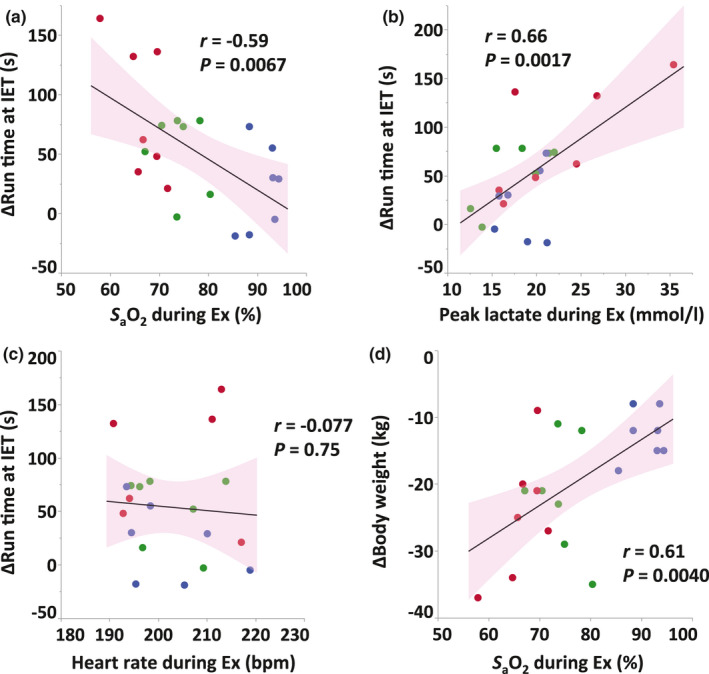
Correlations between *S*_a_O_2_ (a), peak plasma lactate concentration (b), and heart rate (c) during exercise session in the first week and the change in run time at IET after 4 weeks of training, and between *S*_a_O_2_ during exercise session and the change in body weight (d) at IET after 4 weeks of training. Red, green, and blue dots indicate moderate hypoxia (*H16*), mild hypoxia (*H18*), and normoxia (*N21*), respectively. Solid lines indicate regression lines and pink areas indicate 95% confidence intervals.

There were significant correlations between *S*_a_O_2_ during exercise and the changes in run time (*r* = −0.59, *p* = 0.0067; Figure 3a), between peak plasma lactate concentration during exercise and the changes in run time (*r* = 0.66, *p* = 0.0017; Figure 3b), and between *S*_a_O_2_ during exercise and the changes in body weight (*r* = 0.61, *p* = 0.0040; Figure 3d). No significant correlations were observed between heart rate during exercise and the changes in run time (*r* = −0.077, *p* = 0.75; Figure 3c).

## 4 DISCUSSION

The purpose of this study was to determine whether high‐intensity training in moderate and mild hypoxia could improve exercise performance and aerobic capacity to a greater extent than the same training in normoxia. In addition, we sought to determine if two weeks of post‐hypoxic training in normoxia could maintain the benefit of hypoxic training. First, we demonstrated that horses trained in 16% O_2_ enhanced run time and $V_{\dot{V}O2\max}$ at IET more than horses trained in normoxia and that horses trained in 18% O_2_ showed a similar adaptation as *H16*, but there was no statistical significance between *H18* and *N21*. In addition, the acquired hypoxic training effects on performance and aerobic capacity were sustained after 2 weeks of post‐hypoxic training in normoxia.

### 4.1 Training effects on exercise performance and aerobic capacity after hypoxic training

Despite several human studies reported no additional benefits in LLTH training (Faiss et al., 2013), 4 weeks of hypoxic training in *H16* showed greater improvements in run time and $V_{\dot{V}O2\max}$ at IET than normoxic training. Vogt and Hoppeler (2010) stated that there is no clear trend in the effects of LLTH training on performance at sea level and no conclusive recommendations can be made as to which altitude, exposure duration, and exercise intensity might be beneficial. In contrast, (McLean et al., 2014) indicated that enhancements in normoxic performance appear most likely following high‐intensity and short‐term training in hypoxia. Despite our hypoxic settings (*F*_I_O_2_: 16% and 18%) being considered as moderate and/or mild condition for human hypoxic training, horses exercised in *H16* and *H18* experienced severe arterial hypoxemia in this study, and their end‐exercise *S*_a_O_2_ declined to 66.5 ± 1.7% and 74.1 ± 1.7%, respectively (Table 1). Thoroughbred horses often exhibit arterial hypoxemia during high‐intensity exercise even in normoxia mostly due to diffusion limitations in the lungs (Wagner et al., 1989). Previous literature has demonstrated that exercise‐induced arterial hypoxemia also occurs in highly‐trained human athletes during heavy exercise in normoxia and hypoxia, and the end‐exercise *S*_a_O_2_ at *F*_I_O_2_ of 21% was similar to that observed in horses (91 ± 1%), while *S*_a_O_2_ at *F*_I_O_2_ of 17% was not as low as that observed horses (83 ± 1%) (Vogiatzis et al., 2007). These findings suggest that hypoxia may cause more severe exercise‐induced arterial hypoxemia in horses than in humans, and these differences between horses and humans in the severity of exercise‐induced arterial hypoxemia during hypoxic training might induce different training adaptations on performance and aerobic capacity.

In equine studies, whereas (Davie et al., 2017) reported no additional improvements in heart rate and blood lactate concentration during incremental treadmill tests after 6 weeks of moderate‐intensity hypoxic training (3 hypoxic and 3 normoxic sessions/week, total 30 min/session, 15% inspired O_2_), Ohmura et al. (2017) demonstrated that all‐out running for 2–3 min in hypoxia (15.1% inspired O_2_) twice a week for 3 weeks increased $\dot{V}O_{2\max}$ of well‐trained horses in normoxia. Our previous study in horses also showed that 4 weeks of high‐intensity training in hypoxia (100% $\dot{V}O_{2\max}$ 2 min, 3 sessions/week, 15% inspired O_2_) improved performance, $\dot{V}O_{2\max}$, and maximal cardiac output to a greater extent than normoxic training (Mukai et al., 2020). Training programs varied among these studies, including intensity and duration of training, training status of horses (untrained or trained), and hypoxic exposure duration, but these interventions used similar O_2_ concentrations of hypoxic gas. Given that the program of Davie's group, which used a longer duration of training and hypoxic exposure, but lower training intensity, showed no benefit in hypoxic training. The key factors for hypoxic training adaptations in horses may also be high‐intensity and short‐term training as McLean et al. (2014) suggested.

The changes in $V_{\dot{V}O2\max}$ in all groups were very similar to those in run time at IET in our study (Figure 1). While $V_{\dot{V}O2\max}$ is not a direct parameter for running economy, Billat et al. (2003) reported that $V_{\dot{V}O2\max}$ is highly correlated with 10‐km performance time (*r* = −0.86 in men, *r* = −0.95 in women) and $V_{\dot{V}O2\max}$ predicts performance better than $\dot{V}O_{2\max}$ since $V_{\dot{V}O2\max}$ integrates the energy cost of running in addition to $\dot{V}O_{2\max}$. Given that the changes in $\dot{V}O_{2\max}$ after 4 weeks of training were similar in all groups in this study, the improvements in $V_{\dot{V}O2\max}$ may reflect enhanced running economy in submaximal exercise at IET. Several researchers also demonstrated that hypoxic training improves the running economy compared with normoxic training in humans (Katayama et al., 2003; Park et al., 2018; Saunders et al., 2004; Sinex & Chapman, 2015). Barnes and Kilding (2015) stated that altitude acclimatization induces both central and peripheral adaptations that improve oxygen delivery and utilization, mechanisms that may improve running economy. In contrast, Saunders et al. (2004) suggested that the lower aerobic cost of running is not related to ventilation, heart rate, respiratory exchange ratio, or hemoglobin mass. These conflicting results indicate that the mechanism of improved running economy after hypoxic training is unclear and further studies are needed.

$\dot{V}O_{2\max}$, ${\dot{Q}}_{\max}$, and *SV*_max_ at IET increased similarly in all groups throughout the study. In our previous study (Mukai et al., 2020), however, we observed greater $\dot{V}O_{2\max}$, ${\dot{Q}}_{\max}$, and *SV*_max_ in the hypoxic group compared to that of the normoxic group in a similar study design. The causes for these differences are not clear, but the minor differences in the training intensity (100% $\dot{V}O_{2\max}$ vs. 95% $\dot{V}O_{2\max}$), the degree of hypoxia (15% O_2_ vs. 16% or 18% O_2_), and the age of horses (6.5 years vs. 7.9 years) might affect training adaptation on aerobic capacity.

On the other hand, *C*_a‐v_O_2_ was unchanged during the training period in all groups, indicating that the consumed O_2_ in working muscle, that is mitochondrial oxidative capacity, did not change after training. Therefore, the majority of the increase in $\dot{V}O_{2\max}$seems to be induced by the increase in O_2_ delivery. These results suggest that the mitochondrial oxidative capacity is not a limiting factor of $\dot{V}O_{2\max}$in horses and O_2_ delivery is implicated as the primary limitation for $\dot{V}O_{2\max}$, as previously described (Jones & Lindstedt, 1993).

### 4.2 Correlations between *S*_a_O_2_ during exercise session and the changes of variables at IET after 4 weeks of training

We observed a moderate negative correlation (*r* = −0.59) between *S*_a_O_2_ during the training session and the increase in run time at IET, and also a moderate positive correlation (*r* = 0.61) between *S*_a_O_2_ and body weight loss (Figure 3), which suggests that a greater reduction in *S*_a_O_2_ during the exercise session induces a greater improvement in performance and greater weight loss after 4 weeks of training. Given that the lower *S*_a_O_2_ during the training session could simultaneously induce both positive and negative effects, trainers should understand the possibility of hypoxia‐induced weight loss and set an optimal training program for peak racing performance. As monitoring each horse's *S*_a_O_2_ during exercise is not practical at the training track, we recommend monitoring peak lactate concentration instead, which correlated moderately with the change in run time after training (*r* = 0.66), as well as *S*_a_O_2_ (Figure 3).

### 4.3 Effect of hypoxic training on body weight loss

At the same absolute exercise intensity, exercise in hypoxia is perceived as harder (i.e., lower *S*_a_O_2_ and/or higher lactate concentration) and the relative exercise intensity is higher in hypoxia due to the lower $\dot{V}O_{2\max}$than in normoxia (Ohmura et al., 2020). Consequently, hypoxic training can lead to increased energy expenditure, decreased energy intake, and greater body weight loss compared to normoxic training at the same absolute intensity. Furthermore, Katayama et al. (2010) reported that carbohydrate utilization increased during exercise and recovery period in moderate hypoxia compared with normoxic exercise at the same relative intensity. These findings suggest that a shift in substrate utilization may also occur during hypoxic training in horses. Contrary to these results, our previous study demonstrated that well‐trained horses did not reduce their body weight after 3 weeks of high‐intensity training (5 sessions/3 weeks) in hypoxia (Ohmura et al., 2017). These contradictory data indicate that the training status at the beginning of training, as well as the intensity, frequency, and volume of training, may affect the extent of body weight loss during hypoxic training. However, the mechanism of body weight loss during hypoxic training is still unclear and further studies are needed to investigate the relationship between hypoxic training and body weight loss.

### 4.4 Hematological changes with hypoxic training

Consistent with human studies (Roels et al., 2005; Truijens et al., 2003) and our previous study (Mukai et al., 2020), hemoglobin concentrations both at rest and at exhaustion in all groups did not increase after training in our present study. While LHTH and/or LHTL training usually aims to enhance athletic performance by stimulating an increase in serum erythropoietin and erythrocyte volume, only a few well‐controlled LLTH studies on trained or elite athletes have reported increments in hemoglobin concentration (Bonetti et al., 2006), and none have reported any increases in erythrocyte volume and/or hemoglobin mass. Some studies showed that intermittent hypoxic exposure at rest (3 h/day, 5 days/week for 4 weeks at 4000–5500 m altitude) increases serum erythropoietin only immediately after a 3 h hypoxic exposure, but no significant differences were observed in erythrocyte volume or hemoglobin mass compared with the normoxic control (Abellán et al., 2005; Gore et al., 2006). Millet et al. (2010) reported that the minimum daily dose for stimulating erythropoiesis seems to be 12 h/day. These previous reports in humans suggest that the exposure duration to hypoxia (approximately 3 min/session) in this study was too short to increase erythrocyte volume and hemoglobin mass/concentration.

### 4.5 Maintenance of post‐hypoxic training effects

There is contradictory evidence concerning how long the acquired benefits of hypoxic training last after a return to sea level. Pottgiesser et al. (2012) reported that nearly hypoxia‐induced hematological changes observed after 4 weeks of LHTL training may be lost within 9 days, while Brocherie et al. (2015) showed that 14 days of hypoxic training improved repeated sprint performance and hemoglobin mass, with the benefits lasting for at least 3 weeks post‐intervention. Another study also reported that the increase in total hemoglobin mass of elite runners at altitude training camp is stable for 14 days after returning to sea level (Prommer et al., 2010). Consistent with the findings of Brocherie et al. (2015), exercise performance and aerobic capacity after 2 weeks of post‐hypoxic training in normoxia were very similar to those after 4 weeks of hypoxic training in our study, which indicates that most adaptations induced by hypoxic training are maintained after 2 weeks of normoxic training. LLTH training in our study did not induce any changes in hemoglobin concentration throughout the study, which suggests that horses did not gain or lose any benefits from hematological adaptations. This may be one of the reasons that horses maintained their performance and aerobic capacity after post‐hypoxic training in normoxia.

### 4.6 Experimental design of this study

Our study design altered two factors: the $\dot{V}O_{2}$‐based relative training intensity and *F*_I_O_2_. In some human studies, subjects trained at matched relative intensity either in normoxia or hypoxia experience similar adaptations after training (McLean et al., 2014; Truijens et al., 2003). In our design, horses trained in moderate hypoxia showed a greater adaptation in performance without increasing absolute speed. Given that Thoroughbred horses often experience musculoskeletal injuries, we consider that this model of hypoxic training may have benefits of no additional speed/mechanical load. Even if we match the relative training intensity in both normoxia and hypoxia, the absolute speed or mechanical load will decrease in the hypoxic training program, which indicates that we are changing two factors again: the absolute speed/mechanical load and *F*_I_O_2_. In addition, we were concerned about performing two incremental exercise tests for pre‐training measurements in normoxia and for the relative intensity in hypoxia during a relatively short period. Indeed, we understand that further studies that match the relative training intensity in normoxia and hypoxia are needed to clarify the mechanism of hypoxic training in horses.

## 5 CONCLUSION

In this study, we demonstrated that 4 weeks of training in moderate (*H16*), but not mild hypoxia (*H18*) was sufficient to elicit greater improvements in performance and running economy than normoxic training and that the effects of the hypoxic training were maintained over 2 weeks of post‐hypoxic training. Although trainers should monitor weight loss, hypoxic training may be a strategic option for an equine training program without increasing locomotory mechanical stress.

## CONFLICT OF INTEREST

This study was funded by the Japan Racing Association. KM, HO, YT, and TT are employees of the Japan Racing Association.

## AUTHOR CONTRIBUTIONS

Conceptualization: KM, HO, YK, and TT. Investigation: KM, HO, YT, and TT. Formal analysis: KM and TT. Methodology: KM, HO, and TT. Writing—original draft: KM. Writing—review & editing: KM, HO, YT, and YK.

## References

[phy214760-bib-0001] Abellán, R. , Remacha, A. F. , Ventura, R. , Sardà, M. P. , Segura, J. , & Rodríguez, F. A. (2005). Hematologic response to four weeks of intermittent hypobaric hypoxia in highly trained athletes. Haematologica, 90(1), 126–127. 15642679

[phy214760-bib-0002] Barnes, K. R. , & Kilding, A. E. (2015). Strategies to improve running economy. Sports Medicine (Auckland, N. Z.), 45(1), 37–56. 10.1007/s40279-014-0246-y 25164465

[phy214760-bib-0003] Billat, V. , Lepretre, P. M. , Heugas, A. M. , Laurence, M. H. , Salim, D. , & Koralsztein, J. P. (2003). Training and bioenergetic characteristics in elite male and female Kenyan runners. Medicine and Science in Sports and Exercise, 35(2), pp. 297–304; discussion 5–6. 10.1249/01.MSS.0000053556.59992.A9 12569219

[phy214760-bib-0004] Bonetti, D. L. , & Hopkins, W. G. (2009). Sea‐level exercise performance following adaptation to hypoxia: a meta‐analysis. Sports Medicine (Auckland, N. Z.), 39(2), 107–127. 10.2165/00007256-200939020-00002 19203133

[phy214760-bib-0005] Bonetti, D. L. , Hopkins, W. G. , & Kilding, A. E. (2006). High‐intensity kayak performance after adaptation to intermittent hypoxia. International Journal of Sports Physiology and Performance, 1(3), 246–260. 19116438 10.1123/ijspp.1.3.246

[phy214760-bib-0006] Brocherie, F. , Millet, G. P. , Hauser, A. , Steiner, T. , Rysman, J. , Wehrlin, J. P. , & Girard, O. (2015). "Live High‐Train Low and High" Hypoxic Training Improves Team‐Sport Performance. Medicine and Science in Sports and Exercise, 47(10), 2140–2149. 25668402 10.1249/MSS.0000000000000630

[phy214760-bib-0007] Czuba, M. , Waskiewicz, Z. , Zajac, A. , Poprzecki, S. , Cholewa, J. , & Roczniok, R. (2011). The effects of intermittent hypoxic training on aerobic capacity and endurance performance in cyclists. Journal of Sports Science and Medicine, 10(1), 175–183. 24149312 PMC3737917

[phy214760-bib-0008] Davie, A. J. , Evans, D. L. , Hodgson, D. R. , & Rose, R. J. (1999). Effects of muscle glycogen depletion on some metabolic and physiological responses to submaximal treadmill exercise. Canadian Journal of Veterinary Research, 63(4), 241–247. 10534002 PMC1189559

[phy214760-bib-0009] Davie, A. J. , Wen, L. , Cust, A. R. E. , Beavers, R. , Fyfe, T. , & Zhou, S. (2017). The effects of moderate intensity training in a hypoxic environment on transcriptional responses in Thoroughbred horses. Biology Open, 6(7), 1035–1040. 28583927 10.1242/bio.020388 PMC5550905

[phy214760-bib-0010] Desplanches, D. , Hoppeler, H. , Linossier, M. T. , Denis, C. , Claassen, H. , Dormois, D. , Lacour, J. R. , & Geyssant, A. (1993). Effects of training in normoxia and normobaric hypoxia on human muscle ultrastructure. Pflugers Archiv European Journal of Physiology, 425(3–4), 263–267. 8309787 10.1007/BF00374176

[phy214760-bib-0011] Dick, F. W. (1992). Training at altitude in practice. International Journal of Sports Medicine, 13(Suppl 1), S203–S206. 1483776 10.1055/s-2007-1024640

[phy214760-bib-0012] Eaton, M. D. , Evans, D. L. , Hodgson, D. R. , & Rose, R. J. (1995). Maximal accumulated oxygen deficit in thoroughbred horses. Journal of Applied Physiology, 78(4), 1564–1568. 7615470 10.1152/jappl.1995.78.4.1564

[phy214760-bib-0013] Faiss, R. , Girard, O. , & Millet, G. P. (2013). Advancing hypoxic training in team sports: from intermittent hypoxic training to repeated sprint training in hypoxia. British Journal of Sports Medicine, 47(Suppl 1), i45–50. 24282207 10.1136/bjsports-2013-092741 PMC3903143

[phy214760-bib-0014] Fedak, M. A. , Rome, L. , & Seeherman, H. J. (1981). One‐step N₂‐dilution technique for calibrating open‐circuit VO₂ measuring systems. Journal of Applied Physiology, 51(3), 772–776. 7327980 10.1152/jappl.1981.51.3.772

[phy214760-bib-0015] Gore, C. J. , Hahn, A. G. , Aughey, R. J. , Martin, D. T. , Ashenden, M. J. , Clark, S. A. , Garnham, A. P. , Roberts, A. D. , Slater, G. J. , & Mckenna, M. J. (2001). Live high:train low increases muscle buffer capacity and submaximal cycling efficiency. Acta Physiologica Scandinavica, 173(3), 275–286. 11736690 10.1046/j.1365-201X.2001.00906.x

[phy214760-bib-0016] Gore, C. J. , Rodriguez, F. A. , Truijens, M. J. , Townsend, N. E. , Stray‐Gundersen, J. , & Levine, B. D. (2006). Increased serum erythropoietin but not red cell production after 4 wk of intermittent hypobaric hypoxia (4,000–5,500 m). Journal of Applied Physiology, 101(5), 1386–1393. 16794028 10.1152/japplphysiol.00342.2006

[phy214760-bib-0017] Hamlin, M. J. , Marshall, H. C. , Hellemans, J. , Ainslie, P. N. , & Anglem, N. (2010). Effect of intermittent hypoxic training on 20 km time trial and 30 s anaerobic performance. Scandinavian Journal of Medicine and Science in Sports, 20(4), 651–661. 19793215 10.1111/j.1600-0838.2009.00946.x

[phy214760-bib-0018] Hendriksen, I. J. , & Meeuwsen, T. (2003). The effect of intermittent training in hypobaric hypoxia on sea‐level exercise: a cross‐over study in humans. European Journal of Applied Physiology, 88(4–5), 396–403. 12527969 10.1007/s00421-002-0708-z

[phy214760-bib-0019] Jones, J. H. , & Lindstedt, S. L. (1993). Limits to maximal performance. Annual Review of Physiology, 55, 547–569. 10.1146/annurev.ph.55.030193.002555 8466184

[phy214760-bib-0020] Katayama, K. , Goto, K. , Ishida, K. , & Ogita, F. (2010). Substrate utilization during exercise and recovery at moderate altitude. Metabolism, 59(7), 959–966. 20036404 10.1016/j.metabol.2009.10.017

[phy214760-bib-0021] Katayama, K. , Matsuo, H. , Ishida, K. , Mori, S. , & Miyamura, M. (2003). Intermittent hypoxia improves endurance performance and submaximal exercise efficiency. High Altitude Medicine & Biology, 4(3), 291–304. 14561235 10.1089/152702903769192250

[phy214760-bib-0022] Kitaoka, Y. , Endo, Y. , Mukai, K. , Aida, H. , Hiraga, A. , & Hatta, H. (2014). Muscle glycogen breakdown and lactate metabolism during intensive exercise in Thoroughbred horses. The Journal of Physical Fitness and Sports Medicine, 3(4), 451–456.

[phy214760-bib-0023] McLean, B. D. , Gore, C. J. , & Kemp, J. (2014). Application of ‘live low‐train high’ for enhancing normoxic exercise performance in team sport athletes. Sports Medicine (Auckland, N. Z.), 44(9), 1275–1287. 10.1007/s40279-014-0204-8 24849544

[phy214760-bib-0024] Millet, G. P. , Roels, B. , Schmitt, L. , Woorons, X. , & Richalet, J. P. (2010). Combining hypoxic methods for peak performance. Sports Medicine (Auckland, N. Z.), 40(1), 1–25. 10.2165/11317920-000000000-00000 20020784

[phy214760-bib-0025] Mukai, K. , Hiraga, A. , Takahashi, T. , Matsui, A. , Ohmura, H. , Aida, H. , & Jones, James H. (2017). Effects of maintaining different exercise intensities during detraining on aerobic capacity in Thoroughbreds. American Journal of Veterinary Research, 78(2), 215–222. 28140647 10.2460/ajvr.78.2.215

[phy214760-bib-0026] Mukai, K. , Ohmura, H. , Matsui, A. , Aida, H. , Takahashi, T. , & Jones, J. (2020). High‐intensity training in normobaric hypoxia enhances exercise performance and aerobic capacity in Thoroughbred horses: A randomized crossover study. Physiological Reports, 8(10), e14442. 32441408 10.14814/phy2.14442 PMC7243200

[phy214760-bib-0027] Ohmura, H. , Mukai, K. , Matsui, A. , Takahashi, T. , & Jones, J. H. (2020). Cardiopulmonary function during supramaximal exercise in hypoxia, normoxia and hyperoxia in Thoroughbred horses. Journal of Equine Science, 31(4), 67–73. 33376442 10.1294/jes.31.67 PMC7750644

[phy214760-bib-0028] Ohmura, H. , Mukai, K. , Takahashi, T. , Matsui, A. , Hiraga, A. , & Jones, J. H. (2010). Comparison of net anaerobic energy utilisation estimated by plasma lactate accumulation rate and accumulated oxygen deficit in Thoroughbred horses. Equine Veterinary Journal, 42(Suppl 38), 62–69. 10.1111/j.2042-3306.2010.00261.x 21058984

[phy214760-bib-0029] Ohmura, H. , Mukai, K. , Takahashi, Y. , Takahashi, T. , & Jones, J. H. (2017). Hypoxic training increases maximal oxygen consumption in Thoroughbred horses well‐trained in normoxia. Journal of Equine Science, 28(2), 41–45. 28721122 10.1294/jes.28.41 PMC5506448

[phy214760-bib-0030] Park, H. Y. , Shin, C. , & Lim, K. (2018). Intermittent hypoxic training for 6 weeks in 3000 m hypobaric hypoxia conditions enhances exercise economy and aerobic exercise performance in moderately trained swimmers. Biology of sport, 35(1), 49–56. 30237661 10.5114/biolsport.2018.70751 PMC6135977

[phy214760-bib-0031] Pascoe, J. R. , Hiraga, A. , Hobo, S. , Birks, E. K. , Yarbrough, T. B. , Takahashi, T. , Hada, T. , Aida, H. , Steffey, E. P. , & Jones, J. H. (1999). Cardiac output measurements using sonomicrometer crystals on the left ventricle at rest and exercise. Equine Veterinary Journal. Supplement, 30, 148–152. 10.1111/j.2042-3306.1999.tb05206.x 10659240

[phy214760-bib-0032] Pottgiesser, T. , Garvican, L. A. , Martin, D. T. , Featonby, J. M. , Gore, C. J. , & Schumacher, Y. O. (2012). Short‐term hematological effects upon completion of a four‐week simulated altitude camp. International Journal of Sports Physiology and Performance, 7(1), 79–83. 21941010 10.1123/ijspp.7.1.79

[phy214760-bib-0033] Prommer, N. , Thoma, S. , Quecke, L. , Gutekunst, T. , Völzke, C. , Wachsmuth, N. , Niess, A. M. , & Schmidt, W. (2010). Total hemoglobin mass and blood volume of elite Kenyan runners. Medicine and Science in Sports and Exercise, 42(4), 791–797. 19952848 10.1249/MSS.0b013e3181badd67

[phy214760-bib-0034] Puype, J. , Van Proeyen, K. , Raymackers, J. M. , Deldicque, L. , & Hespel, P. (2013). Sprint interval training in hypoxia stimulates glycolytic enzyme activity. Medicine and Science in Sports and Exercise, 45(11), 2166–2174. 23604068 10.1249/MSS.0b013e31829734ae

[phy214760-bib-0035] Robertson, E. Y. , Saunders, P. U. , Pyne, D. B. , Aughey, R. J. , Anson, J. M. , & Gore, C. J. (2010). Reproducibility of performance changes to simulated live high/train low altitude. Medicine and Science in Sports and Exercise, 42(2), 394–401. 19927018 10.1249/MSS.0b013e3181b34b57

[phy214760-bib-0036] Roels, B. , Millet, G. P. , Marcoux, C. J. , Coste, O. , Bentley, D. J. , & Candau, R. B. (2005). Effects of hypoxic interval training on cycling performance. Medicine and Science in Sports and Exercise, 37(1), 138–146. 15632680 10.1249/01.mss.0000150077.30672.88

[phy214760-bib-0037] Saunders, P. U. , Telford, R. D. , Pyne, D. B. , Cunningham, R. B. , Gore, C. J. , Hahn, A. G. , & Hawley, J. A. (2004). Improved running economy in elite runners after 20 days of simulated moderate‐altitude exposure. Journal of Applied Physiology, 96(3), 931–937. 14607850 10.1152/japplphysiol.00725.2003

[phy214760-bib-0038] Sinex, J. A. , & Chapman, R. F. (2015). Hypoxic training methods for improving endurance exercise performance. Journal of Sport and Health Science, 4(4), 325–332.

[phy214760-bib-0039] Truijens, M. J. , Toussaint, H. M. , Dow, J. , & Levine, B. D. (2003). Effect of high‐intensity hypoxic training on sea‐level swimming performances. Journal of Applied Physiology, 94(2), 733–743. 12391107 10.1152/japplphysiol.00079.2002

[phy214760-bib-0040] Vogiatzis, I. , Georgiadou, O. , Koskolou, M. , Athanasopoulos, D. , Kostikas, K. , Golemati, S. , Wagner, H. , Roussos, C. , Wagner, P. D. , & Zakynthinos, S. (2007). Effects of hypoxia on diaphragmatic fatigue in highly trained athletes. Journal of Physiology, 581(Pt 1), 299–308. 10.1113/jphysiol.2006.126136 PMC2075230 17317748

[phy214760-bib-0041] Vogt, M. , & Hoppeler, H. (2010). Is hypoxia training good for muscles and exercise performance? Progress in Cardiovascular Diseases, 52(6), 525–533. 20417346 10.1016/j.pcad.2010.02.013

[phy214760-bib-0042] Vogt, M. , Puntschart, A. , Geiser, J. , Zuleger, C. , Billeter, R. , & Hoppeler, H. (2001). Molecular adaptations in human skeletal muscle to endurance training under simulated hypoxic conditions. Journal of Applied Physiology, 91(1), 173–182. 11408428 10.1152/jappl.2001.91.1.173

[phy214760-bib-0043] Wagner, P. D. , Gillespie, J. R. , Landgren, G. L. , Fedde, M. R. , Jones, B. W. , DeBowes, R. M. , Pieschl, R. l. , & Erickson, H. H. (1989). Mechanism of exercise‐induced hypoxemia in horses. Journal of Applied Physiology, 66(3), 1227–1233. 2496088 10.1152/jappl.1989.66.3.1227

